# How cell death shapes cancer

**DOI:** 10.1038/cddis.2015.20

**Published:** 2015-03-05

**Authors:** V Labi, M Erlacher

**Affiliations:** 1 Max-Delbrück-Center for Molecular Medicine (MDC), Berlin 13125, Germany; 2 Department of Pediatrics and Adolescent Medicine, Division of Pediatric Hematology and Oncology, University Medical Center of Freiburg, Freiburg 79106, Germany; 3 Freiburg Institute for Advanced Studies, University of Freiburg, Freiburg 79104, Germany

## Abstract

Apoptosis has been established as a mechanism of anti-cancer defense. Members of the BCL-2 family are critical mediators of apoptotic cell death in health and disease, often found to be deregulated in cancer and believed to lead to the survival of malignant clones. However, over the years, a number of studies pointed out that a model in which cell death resistance unambiguously acts as a barrier against malignant disease might be too simple. This is based on paradoxical observations made in tumor patients as well as mouse models indicating that apoptosis can indeed drive tumor formation, at least under certain circumstances. One possible explanation for this phenomenon is that apoptosis can promote proliferation critically needed to compensate for cell loss, for example, upon therapy, and to restore tissue homeostasis. However, this, at the same time, can promote tumor development by allowing expansion of selected clones. Usually, tissue resident stem/progenitor cells are a major source for repopulation, some of them potentially carrying (age-, injury- or therapy-induced) genetic aberrations deleterious for the host. Thereby, apoptosis might drive genomic instability by facilitating the emergence of pathologic clones during phases of proliferation and subsequent replication stress-associated DNA damage. Tumorigenesis initiated by repeated cell attrition and repopulation, as confirmed in different genetic models, has parallels in human cancers, exemplified in therapy-induced secondary malignancies and myelodysplastic syndromes in patients with congenital bone marrow failure syndromes. Here, we aim to review evidence in support of the oncogenic role of stress-induced apoptosis.

## Facts

- During cancer development, clonal selection is facilitated by the acquisition of mutations in oncogenes and tumor suppressors and by the selection of 'winner' cells.
- Apoptosis of (pre)-cancerous cells generates vacant niches that potentially become repopulated by more aggressive sub-clones. Thereby, apoptosis increases proliferative pressure and promotes clonal selection, thus driving tumor evolution.
- Dying cells can promote cell division of neighboring cells.

## Open Questions

- Does apoptosis drive malignant transformation in pre-malignant conditions such as therapy-related myelodysplastic syndromes or congenital bone marrow failure syndromes?
- Can resistance to apoptosis delay the risk of (further) malignant transformation within fully established tumors or in pre-malignant tissues?
- How can unnecessary tissue damage and inflammatory response be avoided in tumor patients as well as in patients presenting with premalignant conditions?

Two concepts in the field of tumorigenesis are widely accepted. First, cancer is the result of sequential genetic changes that, eventually, transform normal into malignant cells, a model that has been referred to as multistep carcinogenesis.^1^ Second, specific biological processes have to be deregulated during tumor evolution to enable and sustain tumorigenesis. These processes have been summarized as 'hallmarks of cancer' by Hanahan and Weinberg^2^ in the years 2000 and 2011, respectively, and among those, evasion from cell death is still regarded as an essential mechanism required for malignant transformation and tumor maintenance.

Though every dogma has its day, doubt is the driving force behind scientific progress. In this review, we challenge the current paradigmatic view that increased survival is unambiguously promoting tumorigenesis. We will discuss the role of apoptosis and its deregulation during the induction, progression and maintenance of malignant disease. Finally, we propose to adopt the current view that resistance to cell death constitutes a genuine hallmark of cancer, as we believe that this may actually be limited to certain settings.

## Apoptosis deregulation in cancer

**Figure 1:**
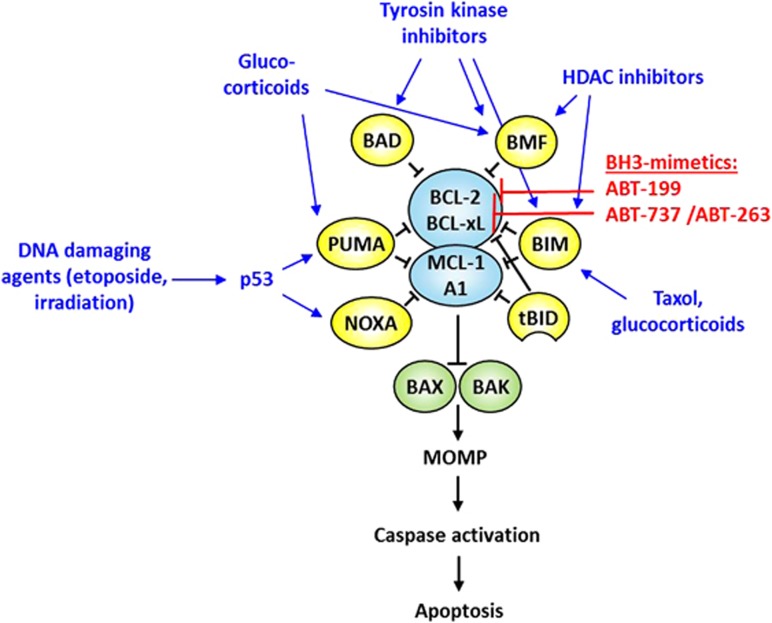
Cytotoxic agents impact the Bcl-2 rheostat. The pro- and anti-apoptotic BCL-2 family proteins closely interact at the mitochondrial membrane and regulate the intrinsic apoptosis pathway. Cellular stress causes activation of pro-apoptotic BCL-2 proteins from the BH3-only sub-group (BIM, PUMA etc.). These bind to and inhibit their anti-apoptotic antagonists (BCL-2, MCL-1 etc.), thus releasing and activating the downstream effectors BAK and BAX. Mitochondrial membrane permeabilization (MOMP) is triggered, and pro-apoptotic molecules released into the cytoplasm activate caspases (in more detail reviewed by Labi *et al.*^120^). Conventional cytotoxic agents interfere with upstream signaling pathways converging at the BCL-2 family level. In contrast, BH3-mimetics directly inhibit pro-survival BCL-2 proteins

Though cells can commit suicide by multiple ways, most cell death in vertebrates is mediated by the mitochondrial (intrinsic) pathway that is initiated by a plethora of signals, such as DNA damage, growth factor deprivation, developmental cues as well as many standard anti-cancer therapies. The initiators of this pathway belong to the BCL-2 family (Figure 1) and the balance between anti- and pro-apoptotic family members, the so-called 'Bcl-2 rheostat' determines whether a cell will live or die an apoptotic death.

On the basis of the 'hallmarks of cancer' concept mentioned above, Green and Evan^3^ proposed that the core changes converting a normal cell into a malignant one might be simply *increased proliferation* coupled to *decreased cell death.* Indeed, it is fully established that tumor cells dampen the apoptotic response, as only defective death prevents their effective elimination by intrinsic anti-cancer mechanisms or anti-cancer therapy.^4^

Progressive counteraction of pro-death signals is a consequence of exogenous as well as (epi)genetic changes in the rich set of factors regulating apoptosis. Though mutations in genes encoding for core components of the cell-death machinery, for example, BCL-2 family proteins, are described in literature, they are not particularly common. One prominent exception occurs in B-cell follicular lymphoma where fusions between BCL-2 and the immunoglobulin heavy chain gene are regularly detected thus raising BCL-2 protein levels.^5, 6, 7^ More commonly, upstream signaling pathways converging at the level of the BCL-2 family are deregulated within tumor cells secondarily leading to an imbalance in the BCL-2 rheostat tilting the balance in favor of survival.

The tumor suppressor gene *TP53* is frequently mutated in human cancer and has early been linked to apoptosis.^8, 9, 10^ Activated upon DNA damage, hypoxia or oncogene activation, TP53 initiates cell cycle arrest and DNA repair or, in irreversibly damaged cells, senescence or apoptosis, with PUMA and NOXA being the main pro-apoptotic target genes.^11, 12, 13, 14, 15^ Loss-of-function mutations in *TP53* result in apoptosis resistance and are frequently associated with advanced tumor stage and poor prognosis.^16, 17^ Besides TP53, other components of the DNA damage checkpoint pathway are frequently inactivated in human cancer (e.g., MDM2, ARF, RB1).^18, 19^ Common mutations in oncogenes causing their growth factor-independent activation can be found in the RAS-signaling pathway, such as mutations in the *RAS* genes itself (i.e., *HRAS*, *NRAS*, *KRAS*) or upstream/downstream components of growth factor signaling pathways (e.g., *FLT3*, *CKIT*, *EGFR*, *PTPN11*, *CBL*, *NF1* etc.).^20^ The main BCL-2 players repressed downstream of these pathways are PUMA, BAD and BIM, BH3-only proteins that are kept inactive by AKT signaling.^21, 22^ In colorectal cancers, the adaptor protein paxillin has been reported to promote survival and chemo-resistance of tumor cells by increasing BCL-2 protein stability and abundance.^23^ The c-MYC-oncogene can induce tumors in its wild-type form and is overexpressed in most human tumor entities.^24^ Paradoxically, at high levels it promotes apoptosis involving BCL-2 family proteins. Many other mutated oncogenes or tumor suppressors have been described to deregulate intrinsic apoptosis signaling but detailed information on their main downstream apoptotic effectors is often lacking.

Besides cell-intrinsic pro-survival signals, support from the environment is critically required for tumor cell survival. For example, chronic lymphocytic leukemia cells survive well in a lymph-node niche *in vivo* but rapidly undergo apoptosis *ex vivo*. This indicates that (i) the downstream apoptosis machinery is usually functional in these cells, and, that (ii) they are ready-to-die and as such strictly dependent on niche-derived pro-survival signals.^25^ Indeed, high levels of the BH3-only proteins BIM, BMF and NOXA were detected in primary human chronic lymphocytic leukemia cells.^26, 27, 28, 29^ Co-culture with feeder or T-helper cells as well as addition of cytokines strongly delays *ex vivo* apoptosis by increasing expression of anti-apoptotic BCL-2 proteins.^25, 30, 31^

## BCL-2 proteins as targets for anti-cancer therapies

Although resistance to cell death is an important feature of cancers, it is certainly not true that tumors are fully resistant to cell death.^32^ If true, anti-cancer therapies would invariably fail. The majority of today's therapeutic regimens aim to directly kill tumor cells and often successfully reduces tumor mass. Depending on the treatment scheme, different but often overlapping pathways are engaged leading to apoptosis and/or necrosis (i.e., DNA damage, oxidative or metabolic stress, and others). Not unexpectedly, clinical trials use the amount of treatment-induced cell death within tumors to predict prognosis and decide on further treatment intensity (i.e., steroid-response in pediatric acute lymphoblastic leukemia).^33^

Most conventional cytotoxic agents act by activating the intrinsic apoptosis pathway (Figure 1). DNA damaging agents (i.e., etoposide or alkylating agents) as well as *γ*-irradiation induce apoptosis by TP53-mediated activation of PUMA and possibly also NOXA.^12, 13^ In contrast, steroids kill acute lymphoblastic leukemia cells by activation of BIM, PUMA and/or BMF,^34^ and imatinib has been shown to kill BCR-ABL-positive chronic lymphocytic leukemia cells in a BIM- and BAD-dependent manner.^35^ Alternatively, apoptosis susceptibility of chronic lymphocytic leukemia cells can be increased by the CXCR4 antagonist plerixafor because of mobilization-dependent loss of survival signals in lymph-node niches.^25, 36^ Thereby, pro-survival signals within tumor cells drop and the BCL-2 rheostat favors induction of apoptosis.

Recently, specific compounds that induce apoptosis directly at the BCL-2 level have been developed (Figure 1). These 'BH3-mimetics' (i.e., ABT-737, ABT-263, ABT-199 and Obatoclax) mimic BH3-activity by binding and inhibiting pro-survival BCL-2 proteins.^37^ They hold big promise for anti-cancer therapy, either alone or in combination with other modalities. Among all pro-survival BCL-2 proteins, only BCL-2, BCL-xL and BCL-W are bound with relevant affinities by ABT-737 and ABT-263.^38, 39^ Accordingly, resistance of tumor cells is commonly caused by high levels of MCL-1 and/or BFL1/A1^40^ and thus targeting these two proteins has become increasingly interesting.

## The apoptosis paradox in tumor development

Complete apoptosis resistance coupled with unleashed proliferation would make any tumor grow to a mass of unbearable size in a very short period of time, a fact inconsistent with the usually long latency of malignant disease. An uncontrollably proliferating cell has to undergo only 40 population doublings until a clinically detectable tumor mass comprising approximately 10^9^ cells appears. This lesion would require only 10 further doublings to produce 10^12^ cells, the maximal tumor size compatible with human life.^41^ As such rapid growth is rare, evolving tumors must be characterized by a dynamic interplay between proliferation, cell death and/or senescence. This feature contributes to intra-clonal heterogeneity of tumors that consist of subpopulations of cells displaying variable rates of death, division and aggressiveness.^42, 43^

As discussed earlier, fully transformed cells might have acquired mutations increasing cell death thresholds and inhibiting their clearance.^44^ But how do apoptosis and acquired apoptosis-resistance actually impact on the process of malignant transformation? Is apoptosis resistance sufficient to transform a cell? When and why do transforming or transformed cells require mechanisms to evade apoptosis? And, are there situations where apoptosis resistance rather reduces the risk of (further) malignant transformation?

The involvement of BCL-2 itself in neoplastic transformation was nailed in 1985 when a translocation juxtaposing the *BCL-2* gene and the immunoglobulin heavy chain gene t(14;18) was regularly found in human follicular lymphoma.^5, 6, 7^ This was the first evidence that some oncogenes rather promote cell survival than stimulate proliferation. Only later we learned that apoptosis inhibition *per se* is hardly ever sufficient to transform a cell, in line with the above-mentioned multi-step carcinogenesis model. *BCL-2* transgenic mice develop tumors only at low penetrance and with long latency,^45^ and only a fraction of all persons harboring the t(14;18) translocation in blood cells subsequently develop follicular lymphoma, and only after a long-lasting latency period.^46^

The tumorigenic potential of BCL-2 becomes only evident when overexpressed in combination with oncogenes such as c-MYC. Though promoting unleashed proliferation, c-MYC can only efficiently immortalize cells in the presence of sufficient pro-survival signals such as those provided by overexpression of BCL-2.^47, 48^ This need arises because high levels of c-MYC drive cell death. Thus, apoptosis is an important barrier to uncontrolled proliferation and a form of tumor surveillance curtailing MYC-driven transformation. Consequent studies demonstrated that c-MYC synergizes with any of the anti-apoptotic BCL-2 proteins in transforming leukocytes in overexpression models *in vivo,*^49^ whereas the dependence appears more selective at the level of endogenous pro-survival proteins.^50^ Consistently, the loss of various pro-apoptotic BH3-only proteins results in acceleration of c-MYC-driven lymphomagenesis.^51, 52, 53^ Similar synergies between c-MYC and anti-apoptotic BCL-2 proteins have been observed in other tissues such as the pancreas or the mammary gland.^54, 55^ Furthermore, apoptosis is not only induced by c-MYC overexpression but also by activity-gain of other oncogenes or the loss of tumor suppressors. However, this is beyond the scope of this review and was discussed elsewhere.^4, 56^

Surprisingly, ample data from human tumors and mouse models actually indicate that the 'simple' view on apoptosis being a key mechanism of anti-cancer defense suffers from oversimplification. Studies on human tumors paradoxically pointed out a strong correlation between high BCL-2 levels and favorable prognosis (Table 1). These observations indicate that BCL-2 overexpression is routinely observed in human tumors and that it can be associated with a less aggressive disease course. Along that line, the increased expression of pro-apoptotic BAX has been correlated with an increased risk of relapse in childhood acute lymphoblastic leukemia.^57^ Gurova *et al.*^58^ demonstrated that clonal expansion of transformed TP53-deficient fibroblasts *in vitro* and in a mouse tumor model was suppressed by BCL-2 overexpression. Intriguingly, BCL-2-overexpressing tumors contained genetically stable cells and were able to restrict the expansion of otherwise rapidly growing and genetically instable TP53-deficient cells. In another study, BAX overexpression in the T-cell lineage enhanced lymphomagenesis in TP53-deficient mice in a dose-dependent manner, and even initiated lymphoma formation on a TP53-proficient background.^59^ In accordance with data discussed before, BAX-driven apoptosis led to increased chromosome instability, and co-expression of BCL-2 was able to delay lymphomagenesis.

In sum, these results support a hypothesis in which a higher rate of apoptosis within a tumor, either at early stage or during progression, or both, might promote genetic instability causing more aggressive disease.

## A farewell from the classic view on the role of apoptosis in cancer

**Figure 2:**
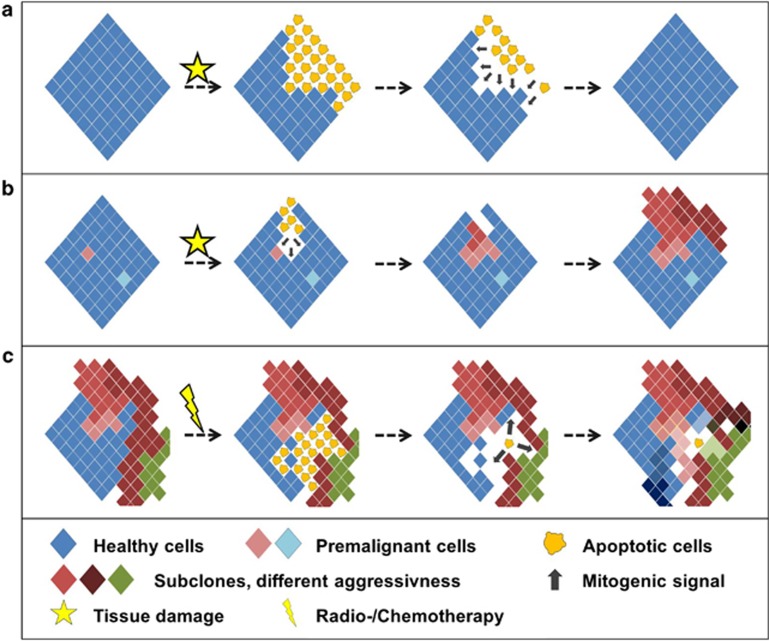
How apoptosis shapes cancer. (**a**) In proliferative tissues, injury is followed by rapid regeneration and restoration of normally sized and shaped structures. In the *Drosophila* wing imaginal disc, apoptotic cells induce competitive proliferation by secretion of mitogenic factors in a caspase-dependent manner. (**b**) In tissues with aberrant cells, tissue injury (e.g. caused by DNA damage in MDS patients) and consecutive proliferation enables outgrowth of more aggressive clones. This fosters malignant transformation. (**c**) Within established tumors, chemo- or radiotherapy induces apoptosis but leads to death-induced proliferation of therapy-surviving cells. Following the generation of space, proliferation is mediated by mitogens derived from apoptotic cells (such as PGE_2_). As proposed in mathematical models, this results in increased sub-clonal variability with a higher risk of tumor progression, chemoresistance and relapse

How could apoptosis promote tumorigenesis? Cancer development can be viewed as a Darwinistic process of somatic cell evolution, whereby initially 'healthy' cells acquire multiple (epi)genetic lesions driving clonal selection. This process is facilitated by the acquisition of mutations in oncogenes and tumor suppressors and by the selection for cells with superior fitness. Under continuous selection pressure, apoptosis could be a major driver of clonal expansion by generating vacant niches (Figure 2). These niches become repopulated by more aggressive sub-clones with certain competitive advantages. In that respect, apoptosis would be a driver of tumor evolution and a hallmark of aggressive disease. This could be especially relevant during early steps of tumorigenesis. Pre-malignant lesions can stably persist for an extended period of time while still too small to be clinically relevant.

Proof-of-principle experiments exploring cell competition and compensatory proliferation in *D. melanogaster* larval development suggest a dual role for apoptosis during early tumorigenesis with the need to dampen intrinsic pro-apoptotic signals to promote tumor cell survival on one hand, and the benefit from the death of surrounding cells on the other hand.^60, 61^ This can be described as a type of Darwinian-like selection that generates 'winner' and 'loser' cells thus leading to long-term outgrowth of certain cells over others. In particular, preventing apoptosis of surrounding wild-type cells impairs the growth of otherwise highly proliferating clones, both in the cases of DMYC-induced super-competition^60^ and *Minute*-induced cell-competition.^61^ It has only recently been shown that this competition relies on TOLL signaling inducing NF*κ*B-dependent apoptosis in 'loser' cells and their subsequent engulfment by 'winner' cells.^62, 63^

Along this line, a natural cell competition has been described for thymic progenitor cells in the mouse. Young cells recently immigrated from bone marrow displace 'older' progenitors already residing in the thymus. The 'older', 'loser' cells, express lower BCL-2 levels and are more susceptible to apoptosis. Consequently, reduction of natural competition in healthy thymic tissue causes T-cell lymphoma.^64, 65^

Mathematical models allow for an approximation of how intrinsic cell properties influence growth dynamics and clonal expansion.^66, 67^ Enderling *et al.*^68^ predict that spontaneous cell death yields a tumor size reduction in the short term, but ultimately enhances tumorigenesis in the long term. They conclude that tumors can remain dormant for long intervals despite constant cellular turnover and that high apoptosis rates perturb the intrinsic tumor dynamics and shift the population towards more aggressive subclones.^69^ Wodarz *et al.*^70^ describe the relation between death rate and the generation of mutant cells within a population after a first wave of clonal expansion. In their mathematical model, they find that less cell death correlates with fewer cell divisions during clonal expansion, thus leading to a less variable cell population. In contrast, high death rates correlate with more cell divisions during expansion causing the appearance of many different mutants (Figure 2). With increasing sub-clonal variability, the risk that individual cells overcome selective barriers (i.e. growth inhibition) and progress towards malignancy increases.^70^ We are putting forward the question whether these *in silico* models on cell death-stimulated tumor progression find their counterparts *in vivo*.

## Death-driven proliferation facilitates tissue regeneration and tumorigenesis

One piece of evidence in favor of these mathematical models comes from two studies on thymic T-cell lymphoma where genetic ablation of the pro-apoptotic BH3-only protein PUMA abolished tumor formation.^71, 72^ In this mouse model, lymphomagenesis is induced by repeated rounds of sub-lethal *γ*-irradiation and strongly accelerated by TP53 deficiency.^73^ Repeated *γ*-irradiation induces a massive wave of apoptosis in the hematopoietic compartment that is dependent on the TP53 target, PUMA.^13^ Initially, these studies aimed at confirming the tumor suppressor potential of PUMA, as was suggested by the fact that its loss accelerated MYC-induced lymphomagenesis.^53, 74^ Unexpectedly, PUMA deficiency protected mice efficiently from thymic lymphoma. Why does deletion of PUMA abrogate lymphoma formation whereas loss of its activator, TP53, does the opposite? Two studies on the competition of hematopoietic progenitors shed light on possibly underlying mechanisms. Hematopoietic stem cells (HSCs) carrying damaged DNA introduced by sub-lethal irradiation can effectively reconstitute myelo-ablated mice. However, they are outcompeted when transplanted in a competitive setting with TP53-deficient HSCs.^75, 76^ As transplantation of lethally irradiated recipients requires HSC expansion for hematopoietic regeneration, it is tempting to speculate that selective pressure during repopulation provides the basis for oncogenic mutations to appear. This theory is backed up by a study using tamoxifen-induced TP53 expression on a TP53-deficient genetic background. When TP53 expression was limited to the time of irradiation, DNA damage led to strong apoptosis of hematopoietic cells and subsequently to cancer development. In contrast, when TP53 expression was only allowed at later time points, no apoptosis was induced by irradiation but also no cancer manifested. Thus, the tumor-preventive function of TP53 is not critical during the acute elimination of damaged cells but rather essential at later time points when cells that survived irradiation despite carrying genetic aberrations drive tumor progression.^77^

The observations in PUMA-deficient mice are consistent with these results and suggest that TP53-dependent apoptosis triggered during an acute DNA damage response is not only irrelevant for tumorigenesis, but even promotes lymphoma formation. This is underlined by the finding that resistance to radiation-induced lymphoma in PUMA-deficient mice can be overcome by PUMA-independent apoptosis induction, that is, by glucocorticoid treatment upon irradiation.^72^ The tumor-initiating cells in this tumor model are hematopoietic stem or progenitor cells, because T-cell specific overexpression of pro-survival Bcl-xL failed to prevent irradiation-induced lymphomagenesis despite protecting thymocytes and peripheral T cells from death whereas mice overexpressing BCL2 throughout hematopoiesis phenocopied PUMA-deficient mice.^71, 72^ Strikingly, in wild-type mice, persisting PUMA- and TP53-dependent apoptosis is still detected 1 week after irradiation specifically in hematopoietic progenitors as compared with more differentiated cells indicating excessive pressure within the proliferating progenitor compartment to compensate for the cell loss.^78^

The association between death-driven proliferation and cancer is best established in mouse models of hepatocellular carcinoma (HCC). In humans, HCC almost invariably develops in the context of chronic liver inflammation that is linked to tissue injury and cell death caused by viral hepatitis, chronic alcohol consumption, excessive hepatosteatosis or environmental toxins.^79^ The regenerative response is accompanied by a release of pro-inflammatory factors by dying hepatocytes and subsequent expansion of un-differentiated precursors such as tissue stem cells. Given the strong impact of apoptosis on HCC development, Qiu *et al.*^80^ investigated the role of PUMA in a mouse model of carcinogen-induced liver cancer. They found that PUMA was activated by JNK1 and critically mediated carcinogen-treatment-induced apoptosis. Importantly, PUMA deficiency decreased the multiplicity and size of emerging tumors.^81^ Two further studies could show that liver-specific Mcl-1 deletion induced spontaneous hepatocyte apoptosis, chronic proliferation and finally caused HCC. Noteworthy, in this mouse model, HCC developed in the absence of carcinogen treatment or detectable inflammation and hepatocytes of HCC-like lesions showed a high degree in genomic instability.^82, 83^

## Mechanisms coupling cell death and proliferation

The discussed mouse models indicate that apoptotic cells promote cell divisions of neighboring cells, a process that can be termed death-driven proliferation, but the underlying signaling events remain elusive.^84^ Only recently, evidence has emerged how apoptotic cells can promote the proliferation of surrounding cells. Planarians regenerate complete individuals from the smallest of body parts upon injury^85^ by a process termed compensatory proliferation.^86, 87^ Apoptosis mediated by pro-death effectors like the caspase-like gene 3 (DjCLg3) is not restricted to the wound but occurs in primarily unaffected tissue and is thus actively involved in driving full restoration of body pieces.^88^ In Hydra, head regeneration after amputation requires secretion of Wnt3 by dying cells, thus initiating *β*-catenin-driven proliferation of surrounding cells. Blocking apoptosis by caspase inhibitors prevents head regeneration and can be overcome by exogenous Wnt3.^89^ In the *Drosophila* wing imaginal disc, a highly proliferative tissue, radiation-induced apoptosis is followed by rapid tissue regeneration to form adult structures of normal size and shape (Figure 2). Mechanistically, activity of the initiator-caspase Dronc in apoptotic cells promotes JNK and Wingless signaling pathways, thus causing the secretion of mitogens Decapentaplegic (Dpp) and Wingless (Wg) to promote tissue regeneration.^90, 91, 92^ Keeping apoptosis-initiated cells artificially alive by inhibiting downstream effector caspases prevented injury-induced death. Persistence of such 'undead' cells resulted in excessive proliferation and hyperplastic overgrowth due to continuous and inappropriate secretion of mitogens. In contrast to proliferating tissues where Dronc-initiated apoptosis induced Dpp and Wg expression, apoptosis-induced proliferation in committed non-dividing photoreceptor neurons in *Drosophila* larvae required activity of the effector caspases DrICE and Dcp-1, which subsequently force cell cycle entry mediated by Hedgehog (Hh) signaling.^93^

Extrapolating to human tumors, the ability of apoptotic cells to actively promote proliferation of surrounding cells, for example by secreting mitogens, might be of major significance. We speculate that cell–cell communication likely couples proliferation and cell death either passively or through signals actively elicited by apoptotic cells. In support of the latter, a recent study in xenotransplanted mice suggests that dying cells directly induce proliferation of neighboring cells. Upon radiotherapy-induced tumor cell apoptosis, caspase-3 activity led to the activation of iPLA_2_ and subsequent release of prostaglandin E_2_ by apoptotic tumor cells and neighboring stroma. Prostaglandin E_2_ in turn served as promoter of tumor cell survival and proliferation.^94^ In this model, the net response to therapy was determined by radiation-induced tumor cell apoptosis and prostaglandin E_2_-driven cell survival and proliferation.

An additional layer of communication between dying and proliferating cells is provided by immune and inflammatory cells. Dying cells activate macrophages, dendritic cells, neutrophils and mast cells that secrete mitogenic cytokines such as IL1, IL6 or TNF*α.*^95^ Thus, next to their major function of immune surveillance,^96^ the immune and inflammatory systems also foster malignant transformation under certain circumstances.^95^

In sum, we propose that understanding the impact of death-driven proliferation on tumorigenesis, either directly or mediated by inflammatory signals, can open a new avenue to improve therapy and potentially prevent cancer development.

## From animal models to human disease

The animal models discussed earlier indicate that too much apoptosis compromises healthy or premalignant tissues by increasing proliferative pressure and clonal selection fostering outgrowth of malignant clones. But do these observations reflect tumorigenesis as it occurs in humans? And if yes, which tissues and cancer types could be affected?

The concept of apoptosis-driven cancer can be applied to therapy-induced secondary tumors that originate from distinct tissues than the primary tumors. These tumors arise as a consequence of genomic instability^97^ likely provoked by repeated cycles of excessive apoptosis and subsequent proliferation during therapy. Typically, they compromise tissues with high regenerative capacity (i.e., breast, intestine, rectum, skin or thyroid gland) and frequently present in the area of previous irradiation.

Adult survivors of childhood cancer have a sixfold increased risk to develop secondary tumors later in life.^98^ This is either due to underlying genetic (e.g., germline mutations in cancer susceptibility genes^99^) and/or environmental factors (e.g., nicotine abuse) that predispose these patients to tumors or to previous therapies including chemotherapy or irradiation. Patients who suffer from a combination of an underlying genetic predisposition and earlier application of chemotherapy or radiation therapy have an excessively high risk to develop secondary tumors.^100^

Bone marrow, a radiosensitive tissue,^101^ frequently gives rise to secondary malignancies as suggested by the above-described mouse model of irradiation-induced lymphomagenesis.^71, 72^ However, thymic lymphomas are rare in humans and do not occur as therapy-induced malignancies. In contrast, in humans, exposure to radio- or chemotherapy rather increases the risk to develop therapy-related myelodysplastic syndromes (MDS).^102^

**Figure 3:**
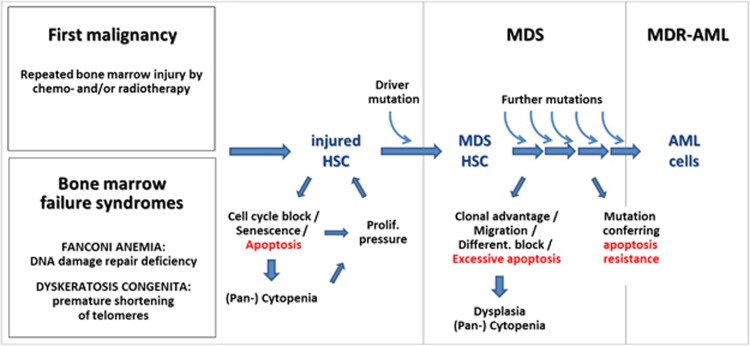
The rise and fall of apoptosis during MDS pathogenesis. Therapy-related myelodysplastic syndrom (MDS) is caused by repeated cycles of radio- or chemoradiotherapy (i.e., including alkylating agents) that lead to bone marrow attrition and subsequent regeneration. In children and adolescents, MDS can develop on the basis of congenital bone marrow failure syndrome such as Fanconi anemia and Dyskeratosis congenita. MDS frequently progresses to MDS-related AML (MDR-AML). The stepwise evolution of MDS is reflected by the FAB classification, which distinguishes between refractory anemia (RA), RA with excess blasts (RAEB), RAEB in transformation (RAEB-T) and MDR-AML

MDS are clonal malignancies originating from defective bone-marrow-derived HSCs, in which critical driver mutations provide them with a selective advantage (Figure 3). This disease is characterized by ineffective hematopoiesis causing peripheral cytopenia(s) and bone marrow dysplasia. Abnormal clonal progenitor cell differentiation and increased susceptibility of immature progenitors to apoptosis underlie these symptoms. The risk to develop MDS increases with age, suggesting that accumulation of genetic damage influences pathogenesis. Exposure to alkylating agents, chemo- or radiotherapy of cancer patients dramatically increases the risk to develop therapy-related MDS (lifetime risk of 2–10%).^102^ MDS has a high propensity to progress to MDS-related AML (MDR-AML). Disease progression is characterized by an increased percentage of bone marrow blast cells and cytogenetic abnormalities (reviewed by Corey *et al.*^102^).

In analogy to therapy-related MDS of adults, children and adolescents can develop secondary MDS and MDR-AML on the basis of congenital bone marrow failure syndromes (Figure 3). These syndromes are caused by gene mutations affecting diverse cellular pathways but all resulting in premature failure of hematopoiesis. In individuals with these congenital conditions, HSCs become prematurely exhausted and are excessively susceptible to apoptosis or senescence.^103^ The most frequent bone marrow failure syndromes are Fanconi anemia, caused by mutations in DNA repair genes, and dyskeratosis congenita, characterized by premature telomere shortening.^103, 104^ Fanconi anemia and dyskeratosis congenita have an inherent risk to transform into MDS, with prevalence of 30–40% and 10–15%, respectively.^105^ Additionally, patients with these syndromes are at risk to develop other malignancies, with those children having the highest risk that previously were subjected to chemotherapeutic agents or irradiation.^105^

Both, therapy-related and secondary MDS are caused by cumulative HSC injury via DNA damage or oxidative stress. In patients with or animal models of bone marrow failure syndromes and low-risk MDS, HSCs are exceptionally susceptible to apoptosis.^106, 107, 108^ This indicates that, in line with the aforementioned animal models, excessive apoptosis generates vacant cell compartments that subsequently are repopulated by more competitive HSCs. Proliferative pressure is further increased by peripheral cytopenias and feedback loops to the bone marrow. Thus, apoptosis might be a major driver of disease progression during early stages of MDS and final transformation to full-blown AML. The pathophysiology of Fanconi anemia, dyskeratosis congenita and therapy-related MDS suggests that chronic HSC apoptosis can be attributed, at least in part, to chronic DNA damage checkpoint signaling, with ATM/ATR, CHK1, CHK2 and TP53 being central players.^109^ These checkpoints preserve genetic stability and act as a barrier to malignant transformation,^110^ thus evolving tumor cells are in need to inactivate them. Accordingly, the amount of apoptotic CD34+ HSCs gradually decreases during further progression to MDR-AML. The pressure to inactivate DNA damage checkpoint signaling is reflected by the fact that therapy-related AML more frequently harbor *TP53* mutations than AML developing *de novo*^111^ and that clones harboring *TP53* mutations are selected during malignant transformation of therapy-related AML.^112^ Similarly, CHK1 and CHK2 are strongly activated in MDS, but almost completely inactivated in MDR-AML cells.^113^

We have learned from animal models of bone marrow failure and MDS as well as patients' subgroups that cells with activated checkpoint signaling display competitive disadvantages. Accordingly, checkpoint abrogation rescues proliferation and survival of HSCs, but also increases the risk of malignant transformation.^109, 114, 115^ What if, instead of checkpoint abrogation, apoptosis would be inhibited in early-stage MDS whereas all other pathways downstream of the DNA damage checkpoint remain active? On the basis of observations made in the murine thymic lymphoma model, we would expect an increase in bone marrow cellularity, thus relaxing proliferative pressure and delaying further transformation from MDS to MDR-AML. Indeed, the first MDS mouse model available supports this concept: Slape and colleagues^116^ recently showed that BCL-2 overexpression in NHD13 mice corrects macrocytic anemia and delays leukemic transformation. Further mouse models will be required to elucidate the role of apoptosis susceptibility or resistance, respectively, for pathogenesis and progression of bone marrow failure syndromes and MDS to AML.

## Perspectives

Moving away from the paradigmatic view prevailing the last decades, the relationship between cell death and cancer gets far more complex than originally anticipated. Beyond doubt, the traditional view that intrinsic death of potentially dangerous cells is preventive to tumor development still holds true in many aspects. However, it disregards the fact that cells are placed in and interact with their environment. At the first glance, the dual function of apoptosis in tumorigenesis is a challenging concept, but these conflicting roles are not incompatible with common beliefs and might also depend on the tissue and the sequence of events during transformation. The balance between proliferation, senescence and death likely adapts during tumor progression. Tumor initiation must be accompanied by the odd survival of single 'initiated' cells that carry driver mutations. Recently, light has been shed on the process of initiation of hematological malignancies. Driver mutations conferring HSCs with selective advantages (i.e., in *DNMT3A*, *JAK2*, *ASXL1*, *TET2* and others) lead to clonal expansion in aged individuals, who do not (yet) suffer from leukemia or MDS.^117^ During further tumor progression, cancer cells frequently respond to their altered state by undergoing programmed cell death and remain highly dependent on certain survival signals from their environment.^118, 119^ Within a growing tumor, apoptosis will preferentially eliminate those sub-clones with the highest apoptosis sensitivity whereas sparing the more resistant cells. Thus, cell death imposes a huge selection pressure favoring clonal expansion of more aggressive sub-clones. Hence, even fully established tumors are rarely completely resistant to apoptosis, and death induced by hypoxia or chemotherapeutics increases proliferative pressure and clonal selection paving the way for therapy-refractory or relapsing cancers.

In light of these findings, it becomes apparent that standard anti-cancer therapies face a dilemma by aiming at inducing tumor cell death. Hence, we believe that we are in need for better treatment strategies to avoid unnecessary tissue damage and inflammatory responses in tumor patients as well as in patients presenting with premalignant conditions such as bone marrow failure or viral hepatitis. We are still only beginning to understand the complex mechanisms involved in tumor development and progression, and thus further research is necessary to understand the contribution of apoptosis in shaping tumors, as a prerequisite to generate a more comprehensive picture on tumorigenesis and allow more effective therapeutic intervention.

## Figures and Tables

**Table 1:**
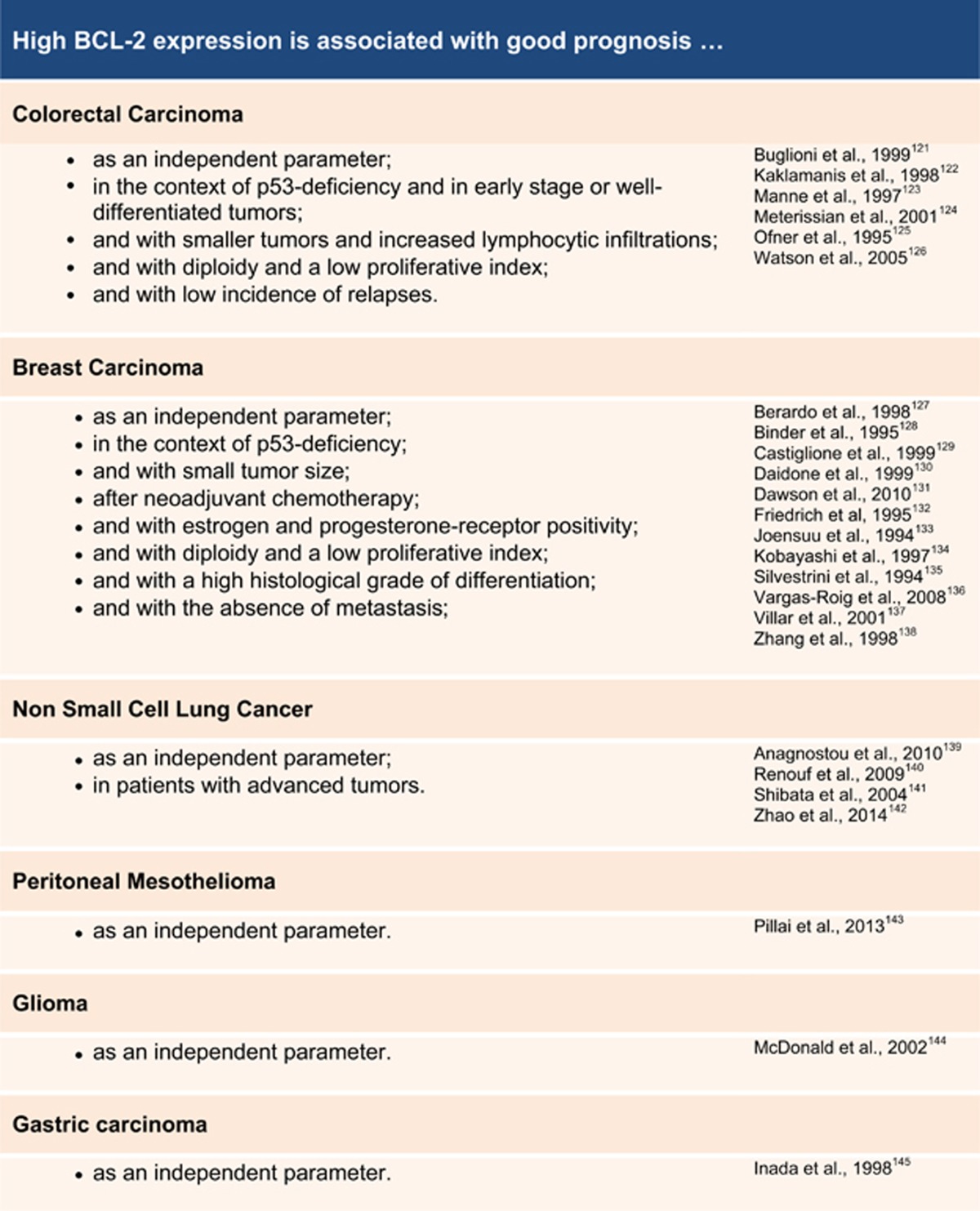
In various tumors, high BCL-2 levels correlate with good prognosis

## Abbreviations

BH3-only: BCL-2 homology domain 3-only
HCC: hepatocellular carcinoma
HSC: hematopoietic stem cell
MDS: myelodysplastic syndrome
MDR-AML: MDS-related acute myeloid leukemia
RA: refractory anemia
RAEB: RA with excess blasts
RAEB-T: RAEB in transformation
